# Safety and efficacy of 2D-fluoroscopy-based iliosacral screw osteosynthesis: results of a retrospective monocentric study

**DOI:** 10.1007/s00068-020-01362-9

**Published:** 2020-04-15

**Authors:** Pol Maria Rommens, Eva Mareike Nolte, Johannes Hopf, Daniel Wagner, Alexander Hofmann, Martin Hessmann

**Affiliations:** 1 Department of Orthopaedics and Traumatology, University Medical Center, Johannes Gutenberg-University, Langenbeckstrasse 1, 55131 Mainz, Germany; 2 Department for Traumatology and Orthopaedics 1, Westpfalz-Clinic Kaiserslautern, Hellmut-Hartert-Straße 1, 67655 Kaiserslautern, Germany; 3 Department of Orthopaedics and Traumatology, Fulda Clinic, Pacelliallee 4, 36043 Fulda, Germany

**Keywords:** Pelvis, Sacral fracture, Iliosacral dislocation, Iliosacral screw, 2D-fluoroscopy, Complications, Malalignment

## Abstract

**Introduction:**

Iliosacral screw osteosynthesis is a well-accepted procedure for stabilization of sacral fractures and iliosacral (fracture) dislocations.

**Materials and Methods:**

In this monocentric study, safety and efficacy of conventional 2D-fluoroscopic-guided iliosacral screw insertion were evaluated.

**Results:**

During a 10-year period (2005–2014), 98 patients between the age of 18 and 65 years received 207 iliosacral screws in 101 procedures. Average patient age was 43.2 years. There were 46 Type B and 40 Type C injuries in the AO/OTA classification, nine patients had a fragility fracture of the pelvis. In three patients, primary radiological data were missing. The indication for surgical treatment was a sacral fracture in 97 patients, a pure iliosacral dislocation in 37 patients and a fracture-dislocation in 31 patients. 70 procedures were performed with the patient in supine position, 31 with the patient in prone position. Surgery was done in a minimal-invasive technique in 76 patients, in 22 patients an open reduction was necessary before screw insertion. 81 patients received a unilateral, 17 patients a bilateral screw osteosynthesis. 199 screws were inserted in S1, only eight screws in S2. 65 patients received two screws unilaterally, ten patients two screws bilaterally. There were no vascular or neurologic complications. During in-hospital stay, there were seven complications, which needed 12 operative revisions: three wound infections, two hematomas, one screw malalignment and one early screw loosening. In 28 patients with 56 iliosacral screws, a pelvic CT-scan was performed during follow-up. A penetration of a cortical layer was diagnosed in 20 of these screws. All penetrations were seen in double screw osteosynthesis of S1. In none of the patients, complaints could be explained by the malalignment of these screws. Five operative revisions were performed during follow-up: two for screw loosening, two for fracture healing problems and one for screw malalignment. Metal removal was performed in 39 patients with 75 screws. 2D-fluoroscopic-guided iliosacral screw osteosynthesis is a safe and efficient procedure in clinical practice.

**Discussion:**

A thorough preoperative evaluation of the morphology of the upper sacrum and careful operative procedure are indispensable. Fluoroscopic views in AP, lateral, inlet and outlet must allow recognition of all anatomical landmarks. The indication for double screw osteosynthesis in S1 should be taken with caution. Screw malalignments do not inevitably correlate with complaints.

## Introduction

Pelvic ring disruptions occur after high-energy trauma. They have different morphologies, depending on the direction of the traumatic force. Rotationally unstable lesions, described as open book and lateral compression injuries, consist of a complete disruption of the anterior and an incomplete disruption of the posterior pelvic ring. Vertically unstable lesions, described as vertical shear injuries, show a complete disruption of the anterior and posterior pelvic ring [1–3]. Soft tissue and hollow organ injuries often accompany fractures and dislocations. Many patients with pelvic ring injuries are severely injured. Emergency management focuses on resuscitation and control of blood loss [4, 5]. The goal of definitive treatment is the restoration of stability and symmetry of the pelvic ring. Bone reconstruction goes parallel with wound management and the restoration of organ function. Operative treatment results in reduced mortality and improved functional outcome [6]. Correct anatomy and adequate stability of the broken posterior pelvis are obtained by open or closed reduction and internal fixation [7, 8]. Several methods of fixation exist and follow different principles of osteosynthesis: bridging the area of instability with plates and screws or bars versus compression of the fracture zone with interfragmentary lag screws. Iliosacral screw osteosynthesis is a reliable method of stabilization of sacral fractures, iliosacral dislocations and fracture-dislocations after high-energy pelvic trauma [9]. It is also successfully used in fragility fractures of the posterior pelvic ring [10–12]. Although widely accepted, the technique is associated with concerns of screw malalignment with damage to vessels or nerves; and with implant loosening. 2D- and 3D-based computer navigations are helpful tools for correct screw placement decreasing the risk of iatrogenic damages [13, 14]. In this monocentric retrospective study, we analyse the safety and efficacy of fluoroscopy-based iliosacral screw osteosynthesis in a large series of patients below the age of 65. The technique is described in detail and the results are compared with those of similar series, in which screw insertion was done similarly or supported by navigation.

### Surgical technique

**Fig. 1:**
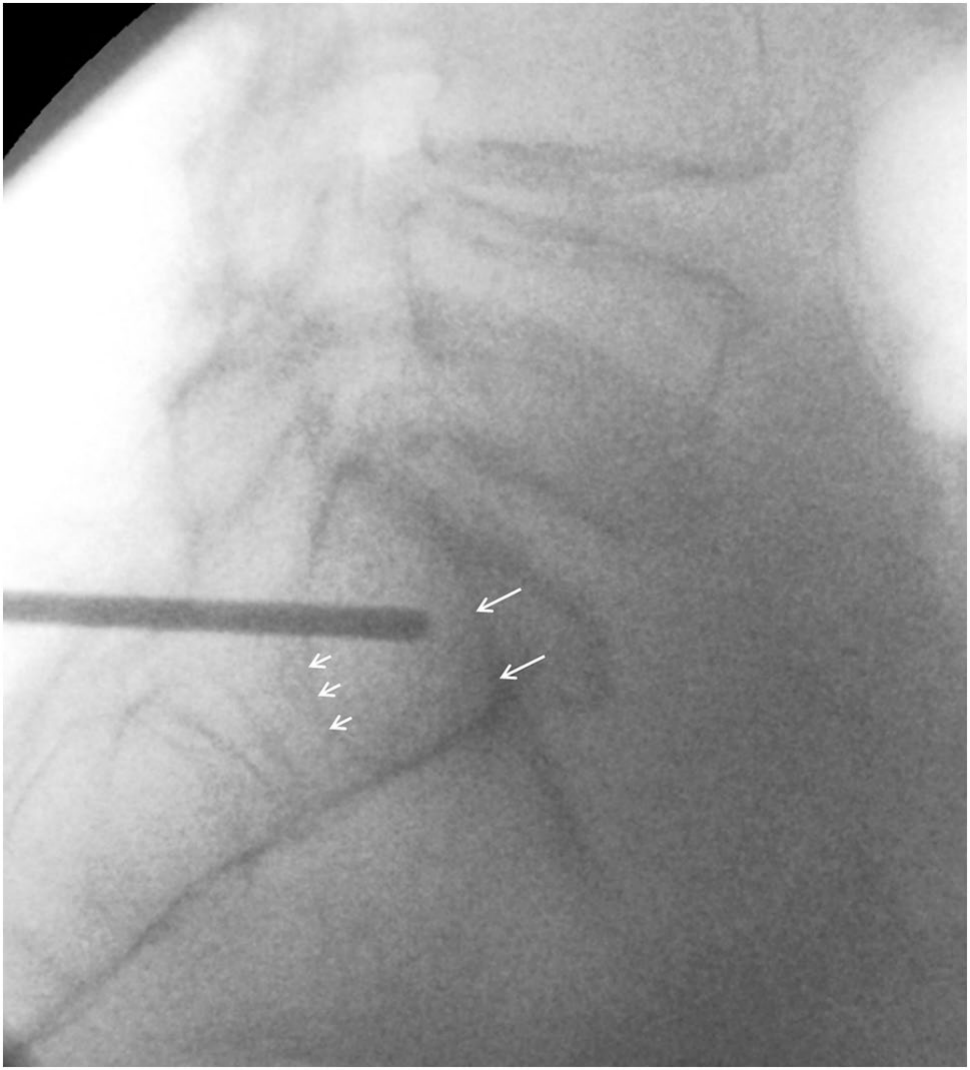
Exact lateral view of the lumbosacral junction. The tip of the drill bit points towards the center of the trapeziform area. Long arrows: alar slope. Short arrows: anterior cortex of S1 root canal

Before surgery, anatomy of the upper sacrum is analyzed with pelvic overviews in AP, inlet and outlet and pelvic CT-data with transverse, coronal and sagittal reconstructions [15–17]. The position of the sacrum is evaluated and the angles of the fluoroscope for exact inlet and outlet views are analyzed. High-quality fluoroscopy images of the posterior pelvis must be available during the surgical intervention. A rectal washout is helpful to prepare the patient on the day before surgery, if applicable. The use of a carbon operation table is recommended. After patient positioning on the operation table and before beginning the procedure, fluoroscopic images in AP, inlet and outlet views are obtained. The following anatomic landmarks of the upper sacrum and the iliosacral joints are identified on the three pelvic overviews: S1 superior endplate, roof of the sacral ala bilaterally, sacroiliac joints bilaterally, neuroforamen S1 and S2 with their curved roofs bilaterally, anterior sacral cortex, sacral canal, canal of S1 and S2 roots. A strict lateral view of the lumbosacral junction is also obtained. The trapeziform area in the S1 body, which corresponds with the safe corridor for screw insertion, is identified. The margins of the trapeziform area are: the sacral alar slope superiorly, the sacral cortex anteriorly, the sacral canal posteriorly, the transition of S1 to S2 and the canal of the S1 root inferiorly (Fig. 1).

**Fig. 2:**
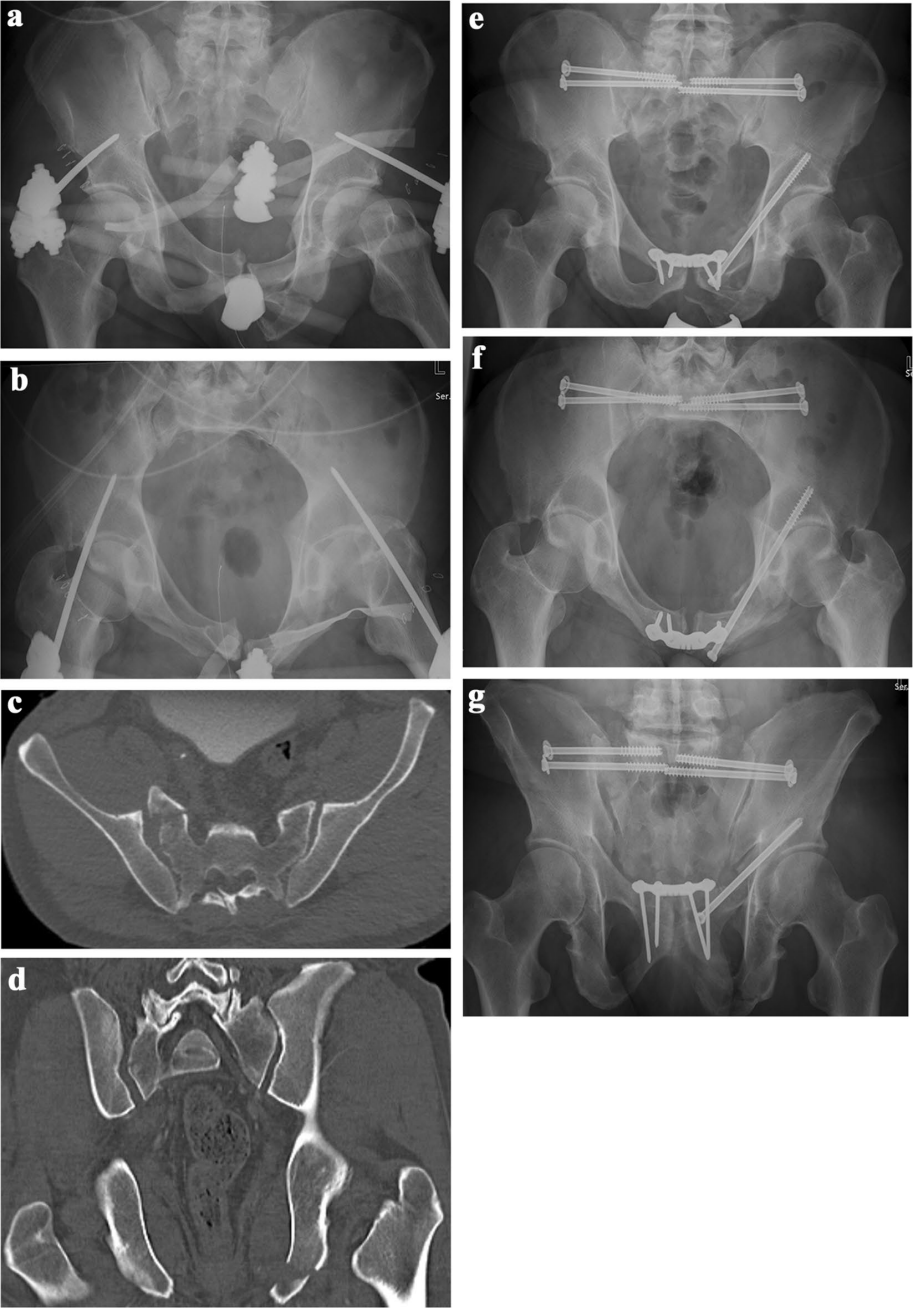
**a** 50-year-old male suffered an unstable pelvic ring injury due a traffic accident with high velocity. AP-view of the pelvis after application of an external fixator. A widening of the right iliosacral joint, a dislocation at the pubic symphysis and left-sided fractures of the superior and inferior pubic rami are visible. **b** Pelvic inlet view. A fracture at the anterior cortex of the right sacral ala and the displacement of the pubic symphysis are visible. **c** Axial CT-slice through the posterior pelvis. There is a widening of both iliosacral joints and a complete fracture through the right sacral ala. **d** Coronal CT reconstruction showing the widening of both iliosacral joints and left-sided fracture of the inferior pubic ramus fracture. **e** Postoperative AP-pelvic overview. Iliosacral joint disruptions and sacral fracture were stabilized with two iliosacral screws on both sides. Dislocation of the pubic symphysis was reduced and stabilized with plate and screw osteosynthesis. Left-sided superior pubic ramus fracture was reduced with a retrograde transpubic screw. There were no postoperative problems. No postoperative CT scan was made. **f** Pelvic inlet view. **g** Pelvic outlet view

The procedure can be performed with the patient in prone or in supine position [18]. The prone position allows for direct access to the sacrum, sacroiliac joint and posterior ilium and enables open reduction, when needed. Due to gravity, the gluteal soft tissues will less interfere with access to the posterior pelvic structures. This is of major advantage in obese persons. In the lateral view, the center of the trapeziform area is identified. Under fluoroscopic control, a vertically directed 2.8 mm diameter drill bit is placed along the skin of the buttock of the injured side, until its tip projects on the center of the trapeziform area (Fig. 1). The drill bit is hold in this position. A small horizontal skin incision is made at the tip of the drill bit. The drill is now placed horizontally and pushed through the gluteal muscles. Under fluoroscopic control, its tip is placed at the ideal insertion point at the outer cortex of the posterior ilium. With a slight hammer blow or a short drilling, the outer cortex is perforated. The correct position of the drill is now controlled under fluoroscopy in AP, inlet and outlet views. The direction of the drill bit is fine-tuned so that it passes the center of the S1 body during the drilling procedure. During the drilling procedure, the tip of the drill bit consecutively perforates the medial cortex of the posterior ilium and the lateral cortex of the sacrum. In regular intervals, the position of the drill bit is controlled in relation to the visible landmarks of S1. Drilling is discontinued when the tip of the drill reaches the opposite sacral ala. In case the opposite sacral ala cannot be reached due to sacral dysmorphism, an as long trajectory as possible is drilled. With a depth gauge, the length of the drill inside the bone is measured. Three cortices (outer and inner cortex of the ilium and lateral cortex of the sacrum) are over-drilled with a cannulated 4.5 mm drill bit, which is glided over the 2.8 mm drill bit. Thread-cutting of the screw trajectory is performed in younger patients. In old patients with osteoporotic bone, no tapping is done to improve the holding power of the screw in the bone. A 7.3 mm or 8 mm cannulated screw of appropriate length is inserted. During insertion, the surgeon can control the distance between washer with screw head and the lateral cortex with an orthograd fluoroscopic view of the posterior ilium; and avoid penetration. The surgeon also feels increasing resistance when the screw head with washer presses directly on the lateral cortex. No compression is obtained when a screw with continuous thread is used; the screw has the function of a positioning screw. Depending on the diameter of the sacral corridor, insertion of one or two screws is possible. In case two screws are inserted in S1, their trajectories are slightly converging in the inlet view to minimize the risk of screw malalignment (Fig. 2a–g). If the second screw is placed in the body of S2, the same steps of the procedure are repeated, taking into account that the dimensions of the sacral corridor of S2 are smaller than those of S1.

In the supine position, the gluteal soft tissues are pushed off towards lateral, which makes access to the posterior ilium more difficult [16]. The patient is placed with the injured side on the margin of a radiolucent table enabling free orientation of the drill bit. The technique of screw insertion is the same as described for the prone position.

## Patients and methods

We reviewed all medical charts of adult patients between 18 and 65 years of age with a posterior pelvic ring injury, who were admitted to the Department of Orthopaedics and Traumatology of the University Medical Center of Mainz, Germany between January 1st 2005 and December 31st 2014 (10-year period). Only patients, who received a fluoroscopy-based iliosacral screw osteosynthesis as part of the stabilization of the pelvic ring were included in the study. Delayed timing of the surgical procedure was not an exclusion criterion. After the procedure, fracture reduction and position of the screws was controlled with conventional a.-p., inlet and outlet views. Only in case of uncertainty or postoperative complaints, a pelvic CT was performed. Epidemiologic data and the following information related to the surgical procedure were collected: trauma mechanism, classification of pelvic ring injury, delay between trauma and intervention, open or closed reduction, level and side of screw insertion, number of screws, quality of screw position, additional stabilization procedures at the pelvic ring, intraoperative and postoperative complications related to the iliosacral screw osteosynthesis, surgical revisions, fracture healing, metal removal. Screw position was rated as published by Gras et al. [19]. An excellent screw position is defined as a screw with complete intraosseous position. An acceptable screw position is defined as a screw which has contact with cortical bone (anterior sacral cortex, neuroforamen, sacral canal). A screw is graded as malaligned when it penetrates cortical bone.

## Results

Ninety-eight patients, younger than 65 years of age, received fluoroscopy-based iliosacral screw osteosynthesis in the mentioned period. There were 56 male (57.1%) and 42 female (42.9%) patients. The average patient age was 43.2 years (median age: 46.0 years). The average age of men was 42.0 and of women 45.4 years. There were 46 traffic accidents, 32 falls and 17 sports or other accidents. In three patients trauma mechanism was not memorable. 59 patients (60.2%) had one or more additional fractures, 21 were polytraumatized (21.4%) and 18 had an isolated pelvic ring injury (18.4%) (Table 1).

**Table 1:** Demographics

Number of patients	98 (100%)
Men	56 (57.1%)
Women	42 (42.9%)
Average (mean) age	43.2 (46.0) years
Average age men	42.0 years
Average age women	45.4 years
Patients with additional fractures	59 (60.2%)
Polytraumatized patients	21 (21.4%)
Patients with monotrauma	18 (18.4%)

There were 46 Type B and 40 Type C injuries in the AO/OTA classification [1, 2]. There were 17 anteroposterior compression (APC), 38 lateral compression (LC) and 31 vertical shear (VS) injuries in the Young-Burgess classification [3]. 9 injuries were classified with the Rommens and Hofmann classification for fragility fractures of the pelvis (FFP) [12]. There were 3 FFP Type II, 3 FFP Type III and 3 FFP Type IV lesions. In three patients, classification was not possible (preoperative conventional X-rays or CT-data not available (Table 2).

**Table 2:** Classification

AO/OTA classification Type B	46
AO/OTA classification Type C	40
Young-Burgess classification LC	38
Young-Burgess classification VS	31
Young-Burgess classification APC	17
Rommens–Hofmann classification FFP Type II	3
Rommens–Hofmann classification FFP Type III	3
Rommens–Hofmann classification FFP Type IV	3
Classification not possible	3

The indications for iliosacral screw osteosynthesis were 97 sacral fractures (63 unilateral and 17 bilateral), 37 isolated iliosacral dislocations (29 unilateral and 4 bilateral) and 31 fracture-dislocations of the iliosacral joint (29 unilateral and 1 bilateral).

In all patients, a concomitant lesion of the anterior pelvic ring was seen. 71 patients suffered pubic rami fractures (47 unilateral and 24 bilateral), while 27 patients suffered a diastasis of the pubic symphysis. In 26 patients, there was a concomitant fracture of the acetabulum, two of them had bilateral fractures (Table 3).

**Table 3:** Fracture description

Sacral fracture unilateral	63
Pure sacroiliac dislocation unilateral	29
Sacroiliac fracture dislocation unilateral	29
Sacral fracture bilateral	17
Pure sacroiliac dislocation bilateral	4
Sacroiliac fracture dislocation bilateral	1
Additional fracture of anterior pelvis	98 (100%)
Unilateral pubic rami	47
Symphysis pubis diastasis	27
Bilateral pubic rami	24
Fracture of the acetabulum unilateral	24
Fracture of the acetabulum unilateral	2

The operative procedure was performed after an average of 4.8 days (median 5.5 days). In 63 patients (64.3%), the screw osteosynthesis was performed as primary procedure, in 21 after a damage control procedure (21.4%) and in 14 after an attempt of conservative treatment (14.3%).

There were 101 operations in these 98 patients. An additional iliosacral screw osteosynthesis was performed in a separate operation in three patients. 70 procedures were performed in supine (69.3%), 31 procedures in prone position (30.7%). In 76 patients, the screw osteosynthesis was performed as a minimal-invasive procedure after closed reduction (77.6%), in 22 patients an open reduction was performed before screw insertion (22.4%).

In total, 207 screws were inserted. In 81 patients, the screw osteosynthesis was done unilaterally (82.7%), in 17 patients bilaterally (17.3%). 199 screws were inserted in S1 (96.1%), only eight screws in S2 (3.9%). 65 patients received two screws unilaterally (66.3%), 16 patients one screw unilaterally (16.3%), ten patients two screws bilaterally (10.2%), six patients one screw bilaterally (6.1%) and one patient received three screws (1.1%) (Table 4).

**Table 4:** Operation technique

Patient in supine position	70 (69.3%)
Patient in prone position	31 (30.7%)
Patients with closed procedure	76 (77.6%)
Patients with open procedure	22 (22.4%)
Number of screw osteosynthesis	207
Patients with 2 screws unilaterally	65 (66.3%)
Patients with 1 screw unilaterally	16 (16.3%)
Patients with 2 screws bilaterally	10 (10.2%)
Patients with 1 screw bilaterally	6 ( 6.1%)
Patients with 3 screws	1 ( 1.1%)
Screws in S1	199 (96.1%)
Screws in S2	8 ( 3.9%)

One or several of the following techniques were used for additional stabilization of the posterior and anterior pelvic ring in 77 patients (78.6%): plate osteosynthesis in 38, retrograde transpubic screw in 29, external fixation in 18, transsacral bar in 6 and lumbopelvic fixation in 2.

There were no iatrogenic vascular or neurologic injuries due to the screw osteosynthesis. Three patients died postoperatively due to traumatic reasons. without a relation to the iliosacral screw osteosynthesis. During in-hospital stay, seven complications (7.1%) were seen, which were related to the screw insertion: three wound infections, two hematomas, one screw malalignment and one early screw loosening. There were 12 operative revisions in five patients, ten of them for infection control in three patients. The other revisions were due to screw malalignment (*n* = 1) and early screw loosening (*n* = 1) (Table 5).

**Table 5:** Complications related to iliosacral screw osteosynthesis. Number and reason of surgical re-interventions

In-hopital death	3 (not related to iliosacral screw osteosynthesis)
Patients with wound infection	3 (3.1%)
Patients with wound haematoma	2 (2.0%)
Number of early surgical revisions	12
Wound debridement	10
Malposition	1
Loosening	1
Number of later surgical revisions	5
Screw loosening	2
Bone healing problem	2
Malposition	1
Metal removal-number of patients	39 (39.8%)
Metal removal-number of screws	75 (36.2%)

Patients could be discharged after an average of 22 days (median 16 days). Follow-up time was 756 days in average (median 502 days). Seventeen patients (17.3%) had an uneventful postoperative course during their in-hospital stay, but did not show up for follow-up. All other patients received conventional pelvic overviews after 3, 6 and 12 weeks. Postoperatively, a pelvic CT-scan was not carried out unless patients had complaints, which could be related to the iliosacral screw osteosynthesis. In 28 patients (28.6%), with 56 iliosacral screws (27.0%), a pelvic CT-scan was performed postoperatively or during follow-up. On these CT-images, a penetration of cortical layer was diagnosed in 20 screws. In none of these screws, cortical penetration was visible on conventional X-rays. All penetrations were seen in double screw osteosynthesis of S1. In none of the patients, the complaints of the patients could be explained by the malaligned screws. Five late operative revisions (5 of 101 procedures = 5%) were performed during follow-up: two because of screw loosening (2% of procedures and 1% of screws), two because of bone healing problems (2%) and one because of malreduction (1%). Metal removal was performed in 39 patients (39.8%) with 75 screws (36.2%) (Table 5).

## Discussion

**Fig. 3:**
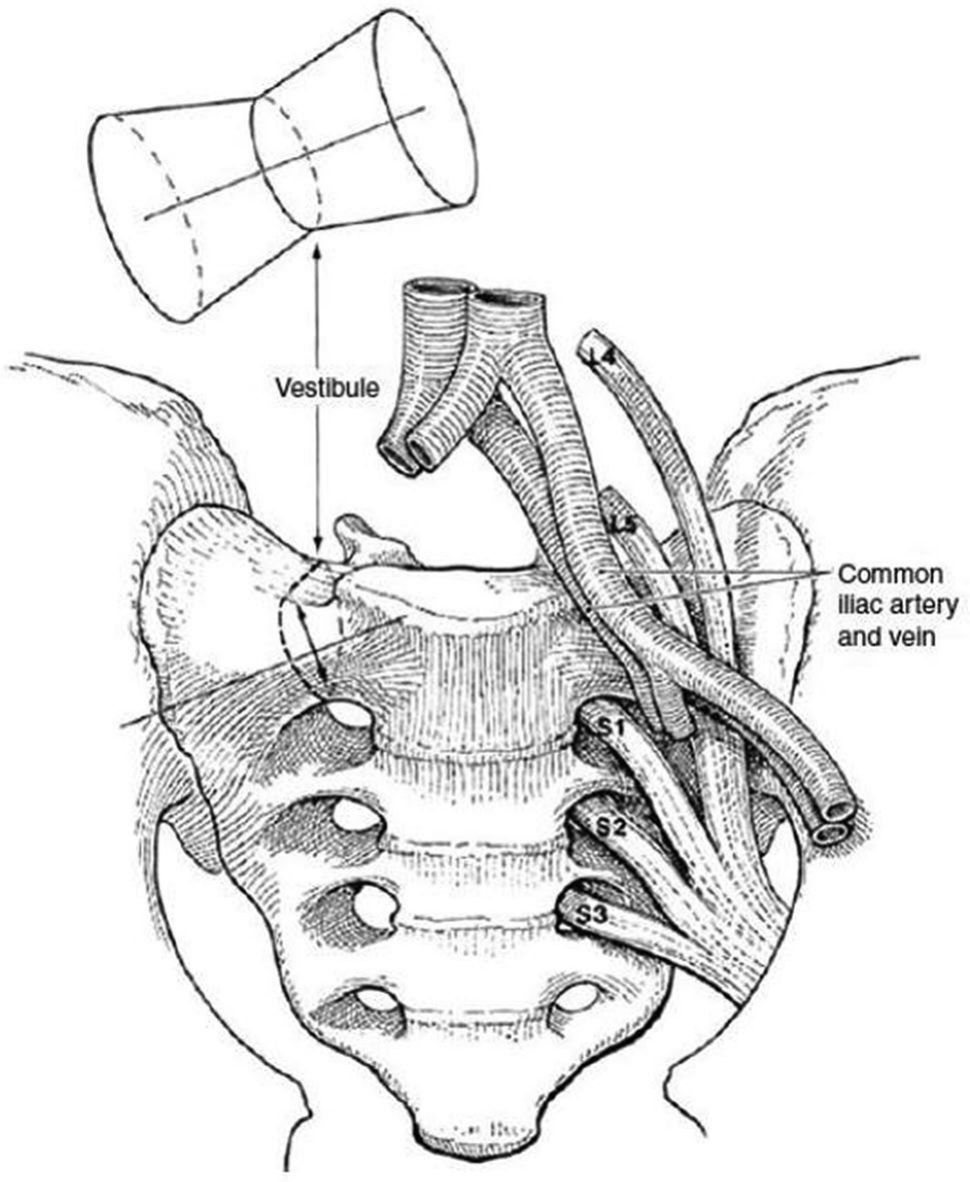
Bone area for iliosacral screw placement has the form of a diabolo with the vestibule being its marrowest passage. It is consistently ovoid in shape and extends from the roof of the S1 neuroforamen to the alar slope. The vestibule always points towards anterior and superior (from Carlson et al. [22])

**Fig. 4:**
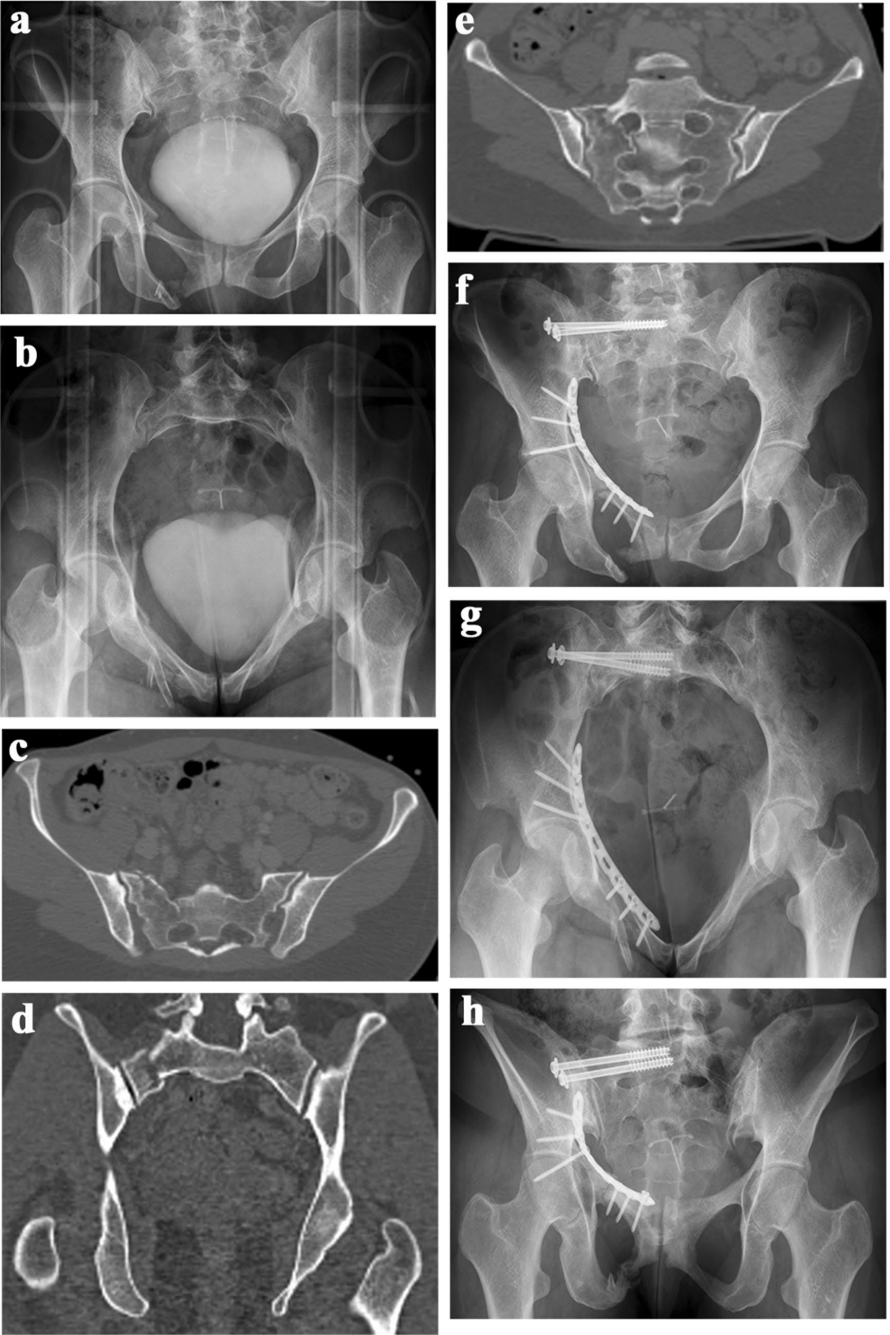
**a** 44-year-old female suffered an unstable pelvic fracture after a fall from 7 m of height. AP-pelvic overview shows displaced superior and inferior pubic rami fractures. **b** Pelvic inlet view shows a right-sided sacral ala fracture and the right-sided superior and inferior pubic rami fractures. **c** Axial CT-slice through the posterior pelvis. There is a right-sided sacral fracture. **d** Coronal CT-slice through the posterior pelvis. The disploaced right.sided sacral fracture is clearly visible. **e** CT-reconstruction through the longitudinal axis of the sacrum. The sacral fracture runs through the neuroforamina S1 and S2. Dysmorphism of the upper part of the sacrum can be recognized. **f** Postoperative AP-view of the pelvis. The sacral fracture was stabilized with two iliosacral screws in S1, the superior pubic ramus fracture was stabilized with a buttress plate through the modified Stoppa approach. There were no postoperative problems. No postoperative CT scan was performed. **g** Pelvic inlet view. **h** Pelvic outlet view

Iliosacral screw osteosynthesis is a widely used and accepted stabilisation technique for instabilities of the posterior pelvic ring. The technique was first described in the 1980s [7, 8]. Biomechanical studies have shown that the obtained stability is equivalent or superior to other stabilisation methods [20]. Van Zwienen et al. proved that a second iliosacral screw in S1 improves rotational stiffness and load to failure [21]. Kraemer et al. calculated that iliosacral screws reaching to the sacral body with a long thread are more stable than short screws, which only reach into the sacral ala [22]. The challenging part of the procedure is the correct placement of the screw(s) within the trabecular bone of S1 and/or S2. The narrowest part of the corridor is the area above the neuroforamen S1 or between the neuroforamina S1 and S2. Carlson et al. introduced the vestibule concept. The safe corridor for screw placement in S1 and S2 has the shape of a diabolo with the vestibule as its narrowest part (Fig. 3) [23]. Depending of the morphology of the upper sacrum and the location of the fracture, the screws are inserted in the coronal plane or oblique. Optimally, the screws are placed perpendicular to the plane of instability: in the coronal plane in vertical sacral fractures and oblique in iliosacral dislocations, fracture-dislocations or sacral dysmorphism (Fig. 4a–h). Oblique screws are directed from inferior-posterior to superior-anterior. In sacral dysmorphism, the S1-corridor is smaller than the S2-corridor [24]. Preoperative analysis and planning are indispensable for safe screw placement. On conventional X-rays and axial, coronal and oblique CT-pictures, the individual anatomy of the upper sacrum and ideal pathway of the screws must be determined, for which planning tools and recommendations exist [15, 25–27].

In this monocentric retrospective study, we reviewed the safety and efficacy of iliosacral screw osteosynthesis in 98 patients. 101 procedures and 207 screw insertions were analysed. We excluded procedures performed in patients above 65 years of age because of the different bone density in the sacrum of older persons. Bone density influences the holding power of the screws and lower bone density enhances the risk of screw loosening [28].

A minority of patients suffered an isolated injury of the pelvic ring (18.4%), more than 80% of the patients had concomitant lesions or were polytraumatized. Only nine patients (9.2%) suffered a low-energy trauma. All patients had a combination of an anterior and posterior pelvic instability. The distribution of high-energy injuries was nearly equal between Type B and Type C of the AO/OTA classification. There were more LC and VS than APC injuries in the Young-Burgess classification. Iliosacral screw osteosynthesis was additionally performed in many rotationally unstable but vertically stable pelvic ring injuries, which had been stabilized with an anterior fixation. In their biomechanical study, Simonian et al. proved that combined fixation of the anterior and posterior pelvic ring obtains the highest stability in APC injuries [29]. Dujardin et al. reported less micromotion at the sacroiliac joint in APC injuries, when anterior plate fixation was combined with iliosacral screw fixation [30]. In our series, operative stabilization of the anterior pelvic ring was performed in more than 75% of patients.

Iliosacral screw insertion was not done on the day of trauma, but after an average of 4.8 days. Iliosacral screw insertion in an emergency situation has a higher risk of screw malalignment with iatrogenic vascular or neurologic damage. In nearly two thirds of the patients, the procedure was performed as first and definitive intervention, only in one fifth after a damage control procedure. Non-emergent intervention after a few days gives the surgical team the opportunity of optimal analysis of radiologic images and best preparation of the patient. A minority of interventions was done with the patient in prone position (*n* = 31, 30.7%). In 22 of them (71.0%), open reduction was performed before internal fixation. The prone position is more cumbersome for the anaesthetist and takes 20 additional minutes for turning and correct positioning of the patient [31].

**Fig. 5:**
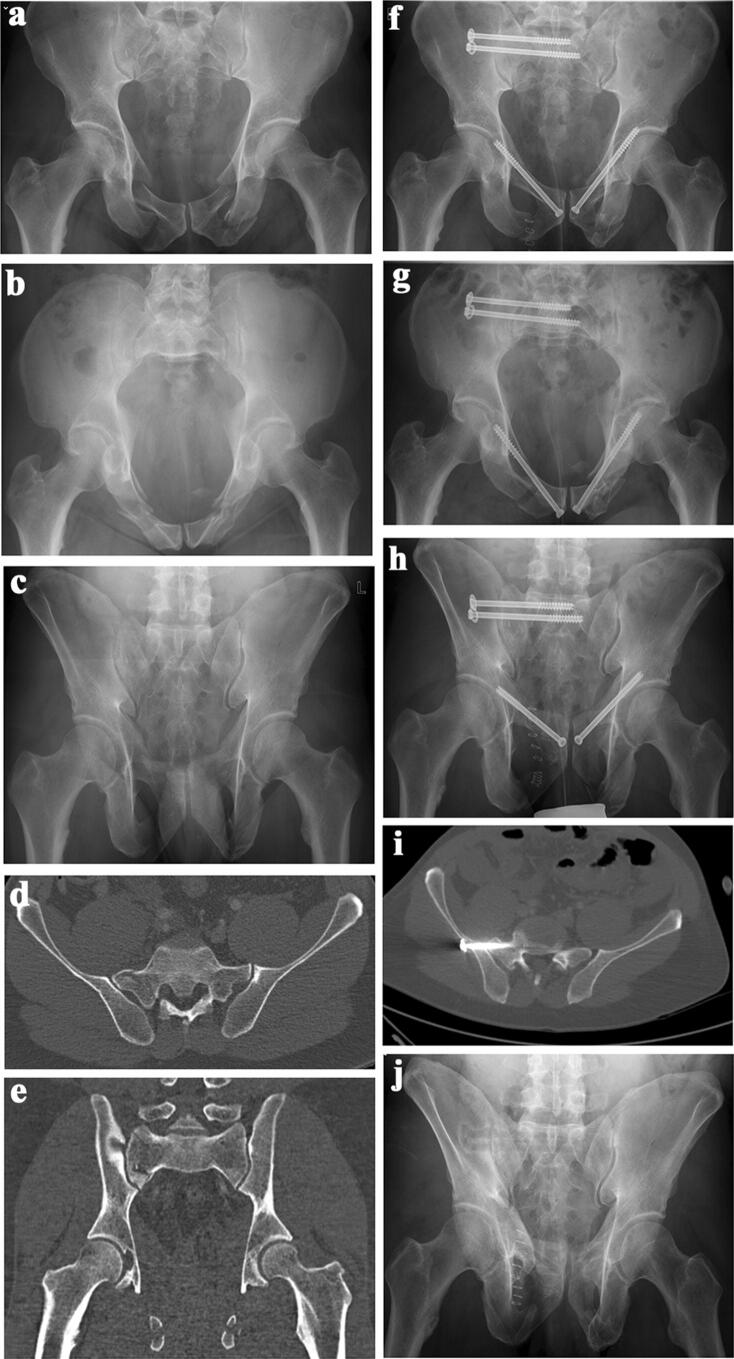
**a** 52-year-old motorcycle driver was hit by a car. AP-pelvic overview showing bilateral superior and inferior pubic rami fracture. A fracture of the posterior pelvis is not visible. **b** Pelvic inlet view. **c** Pelvic outlet view. There is a right-sided sacral fracture running through the neuroforamen S1. **d** CT-slice through the posterior pelvis. A complete fracture of the right sacral ala is visible. The upper sacrum is dysmorphic. **e** Coronal CT-reconstruction showing the right-sided sacral fracture. **f** Postoperative AP-pelvic overview. The sacral fracture was stabilized with two iliosacral screws. The superior pubic rami fractures were stabilized with two retrograde transpubic screws. **g** Pelvic inlet view. **h** Pelvic outlet view. **i** CT-scan through the posterior pelvis. One screw is perforating the anterior cortex of the ilium and lateral sacrum at the iliosacral joint. **j** Pelvic outlet view after removal of the implants

Iliosacral screw placement is regarded as a challenging procedure with the risk of damaging neurological and/or vascular structures. Several technologies have been developed to enhance the accuracy of iliosacral screw placement: planning tools [15, 26, 32, 33], 3D-fluoroscopic navigation [34], CT-based navigation [35, 36] and robot-assisted navigation [37]. Several authors comparing 2D-fluoroscopy with computer navigation prefer the navigation techniques, although screw perforations have been documented between 0 and 22.6% with the navigation technique [36, 38]. In a recent study conducted by Berger-Groch et al. 100 2D-navigated procedures were compared with 36 conventional fluororoscopy-based procedures. There were similar rates of malpositioning, but the radiation exposure was reduced by half when using computer navigation [39]. Other authors mention higher radiation doses for the patient when using computer navigation [40, 41]. Most important is the rate of intra- and postoperative complications due to the procedure. In a large prospective study conducted in 23 German Level I trauma centers, the number of complications did not differ between the conventional 2D-fluoroscopy and the 3D-navigation technique [42]. In our series, early revision surgery due to surgery-related complications was needed in five patients (5.1%). None of the revisions was performed due to neurologic or vascular problems. In 70 patients without any complaints, we did not see the necessity of performing a postoperative pelvic CT-scan. In 28 patients with 56 iliosacral screws, we performed a pelvic CT-scan because of complaints in the pelvic region during follow-up. A cortical perforation was seen in 20 screws, but none of them explained the symptoms of the patients (Fig. 5a–j). In high-energy trauma, persisting pain is frequently seen and may be related to traumatic neurological or soft tissue damage, scar tissue formation or instability [43]. All cases of cortex perforation were seen in patients, who received two unilateral screws in S1. This finding is supported by other literature data. As a result of their anatomical study, Ebraheim et al. concluded that unilateral double screw insertion in S1 has a higher risk of malalignment of the second screw [44]. In their retrospective study on 82 patients with 147 iliosacral screw insertions, Grossterlinden et al. also found that the insertion of two unilateral screws into S1 was associated with a higher screw misplacement rate [45]. In a series of 77 patients, Khaled et al. did not find a different outcome with the addition of a second screw [46]. On the other hand, an additional screw in S1 significantly enhances stability and one iliosacral screw in S1 may not be sufficient in very unstable fracture patterns [21]. In their retrospective study on 62 vertically unstable pelvic fractures, Griffin et al. found four failures of fixation, all of them in vertical sacral fractures [47]. The indication for a second unilateral S1 screw should, therefore, be taken after a thorough analysis of the morphology of the upper sacrum and the fracture pattern of the posterior pelvic ring. In B-type lesions, in which the tension band function of the posterior ligaments is preserved, we believe that the insertion of one S1 screw is sufficient. In C-type lesions, which are vertically unstable, the insertion of two unilateral screws in S1 is preferable, provided that a large corridor is available. In case of a small corridor or dysmorphic sacrum, the insertion of only one S1 screw is safer. An additional screw in S2 or an additional tension band plate or internal fixator is then recommended to obtain the required stability [45]. In adult patients, screws which reach into the sacral body with its high bone mineral density provide sufficient stiffness [22].

Metal removal was done in 39.8% of patients and 36.2% of screws. This high number cannot be explained by complaints due to the implants, but rather by the wish of the patients. In other series, metal removal was only performed in case of implant-related complaints and gave good outcome [48].

We conclude that 2D-fluoroscopy based iliosacral screw osteosynthesis is a safe and reliable procedure for internal fixation of posterior pelvic ring lesions. In an environment, where computer-assisted navigation is not available, 2D-fluoroscopy remains a good alternative. The operation must be prepared through meticulous analysis of the morphology of the upper sacrum on conventional X-rays and CT-data. Although there may exist a higher risk of cortical penetration, this did not correlate with complaints of the patient in our series.

This study has several limitations. After surgery, a CT-scan of the pelvis was merely performed in patients with complaints. This was in a minority of patients (28.6%) and screws (27.0%). Because all patients had a preoperative CT-scan of the pelvis, intraoperative fluoroscopy as well as pre- and postoperative conventional X-rays, we refrained from taking a postoperative CT-scan in every patient to avoid additional radiation exposure. We assumed that the position of the screws was correct in all patients, who did not express any complaints. The malalignment rate following the definition of Gras et al. [19] was calculated with 9.7%. Although without any clinical significance, there may have been additional screws with cortical perforation in the patients, who did not have a postoperative pelvic CT-scan. We did not measure fluoroscopy time of iliosacral screw insertion. In 77 patients (78.6%), an additional anterior stabilization was performed in the same operative procedure, during which fluoroscopy was also done. The exact time of fluoroscopy of the iliosacral screw insertion only could not be distracted from the overall fluoroscopy time in this retrospective study. We did not perform a comparative study with 2D- or 3D-assisted computer navigation as we did not have the soft- and hardware for intraoperative 3D-imaging available in our institution. We did not perform an outcome study of the involved patients, as we focused on the feasibility and safety of the surgical technique. Outcome is depending on multiple factors, of which iliosacral screw osteosynthesis in only one [49]. Despite of these limitations, we still believe that these data are an important addition to the already available literature on this subject.

## Conclusion

2-D fluoroscopic guided iliosacral screw osteosynthesis is a safe and efficient procedure for fixation of posterior pelvic fractures and dislocations. Indispensable condition is the thorough preoperative analysis of the morphology of the upper sacrum, the identification of safe corridors and the recognition of anatomical landmarks in the different fluoroscopic views. Screws with cortical perforation may be clinically silent and not responsible for postoperative complaints of the patient.
